# Abnormal Liver Enzymes during the First Two Months of Liver Transplantation

**Published:** 2015-05-01

**Authors:** B Geramizadeh

**Affiliations:** Transplant Research Center, Department of Pathology, Shiraz University of Medical Sciences, Shiraz, Iran

A 22-year-old woman, known case of autoimmune hepatitis, underwent liver transplantation. She discharged from the hospital in good condition. After 50 days, she presented with abnormal liver enzyme (ALT=117 IU/L, AST=167 IU/L). Laboratory investigation showed positive IgM anti-CMV antibody. Anti-VCA (EBV) IgM was negative. Liver biopsy was performed (Fig 1). All the bacterial and fungal cultures were negative.

## WHAT IS YOUR DIAGNOSIS?

**Diagnosis**: Post-Liver Transplantation Cytomegalovirus Hepatitis

The differential diagnosis of liver dysfunction after orthotopic liver transplantation can be challenging. Cytomegalovirus (CMV) hepatitis is one possibility [1]. It is not common and occurs in 2.4% of liver transplant patients [2]. The incidence of CMV infection after liver transplantation depends on several factors including the available diagnostic tools, donor and recipient sero-status, the peri-operative prophylaxis, and anti-rejection and immunosuppressive regimen used [3].

Diagnosis of CMV hepatitis after liver transplantation can be made by measuring IgM anti-CMV antibody, molecular methods such as real-time PCR, virus culture and liver biopsy. Liver biopsy is the gold-standard test for the diagnosis CMV hepatitis. The diagnosis is made by detecting viral CMV inclusion bodies in H&E and immunohistochemical stating (Fig 2).

**Figure 1:**
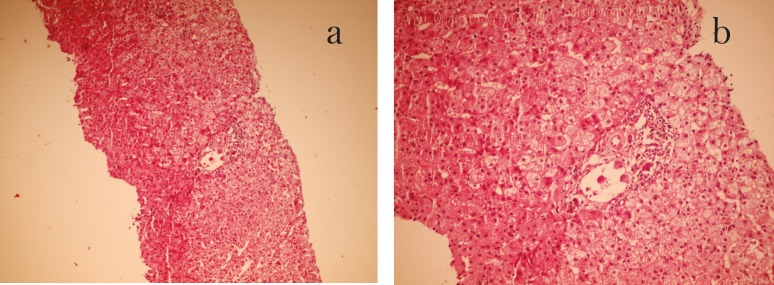
Sections from transplanted liver needle biopsy 50 days after the transplantation. a) Low magnification (×100), b) high magnification (×400) H&E staining

**Figure 2:**
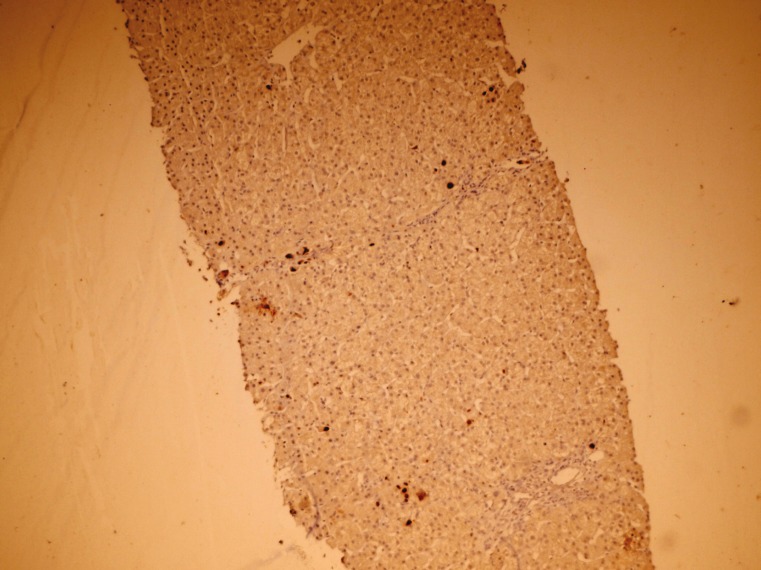
Immunohistochemical staining of sections from the liver needle biopsy showing CMV inclusion bodies
